# Miscellaneous Notices

**Published:** 1843-12

**Authors:** 


### Miscellaneous Notices.

*Our Work.*—The Boston Medical and Surgical Journal, of October 25th, has the following remarks:

"American Journal and Library of Dental Science.—This discreetly conducted periodical, seems to be not only well sustained, but highly prized by the profession.

"To operating dentists, the Journal must be of the very highest value, as it furnishes them with every known improvement in the art from all respectable sources. Our interest in the success of the American Society of Dental Surgeons, and their writings, is unabated."

For these kind expressions, on the part of the editor of the Boston Medical and Surgical Journal, whose approbation is the more gratifying because never bestowed in insincerity, and rarely in profusion, we feel a becoming gratitude. The approbation of the best Medical Journal in New England, frequently and warmly reiterated, shall be improved as an encouragement to continue our exertions in the cause of dental science.

Nor shall we relax our efforts because a few individuals in our profession, regret the establishment of the Society and its Journal, on the ground that it gives publicity to the secrets of the art, and therefore greatly augments the numbers of its professors.

True, the Society and the Journal are alike calculated to communicate to *many*, the useful knowledge which was formerly confined to but *few*. But what is the consequence? Plainly this:—that the profession at large, as well as the public, are greatly benefitted; although a few individuals may be prevented from enjoying a monopoly to the permanent detriment of the majority of mankind.

And what will be the necessary consequence of this diffusion of knowledge in the dental art, and of this augmentation of dental practitioners? Will it not be the elevation of the profession, from isolated quackery to organized and co-operative science; and will not the army of ambitious adventurers who have recently assumed the functions of our art, furnish many noble imitators of Greenwood, Hudson, Cartwright, Parmly, Nasmyth and Brewster, who have contributed the zeal, the perseverance, and the activity of genius, to the welfare of mankind.

If, at this moment of time, there shall be found to be an excessive influx of young artists into our ranks, competing vigorously for the public patronage and favor, both the public and the profession will realize immense advantage from this competition, in years to come. The knowledge of the art of dental practice, and of the science of operative dental surgery, will be every where so widely diffused, that the humblest citizen of our republic will be able to distinguish between the catch-penny quack and the genuine artist, who deserves, as he will receive, the blessings of his fellow men.

This happy result of wide spread knowledge in our art, is already beginning to be realized in the southern section of the United States, where the difference between a good dental operator and a charlatan is very quickly detected. The same is true in certain sections of the northern and western states, but not to the same extent as at the south, because these latter regions have not been so long and so thoroughly canvassed by all sorts of dental operators; nor, perhaps, is the necessity of dental services so frequent in the higher as in the lower latitudes.

Scarcely a day passes in which we do not obtain new evidences of the increased confidence which the public reposes in good dental operations, and also in the decisions of the American Society of Dental Surgeons, on the subject of those individuals deemed worthy of its fraternity. There are some, peradventure, among its members, as in all other associations, who little deserve the distinction which membership in this society is calculated to bestow; but the great majority of the associates are found not only to deserve but to command the confidence of the public.

As something like twenty or thirty new members are created at each successive annual meeting of the Society, it will soon assume a formidable aspect, and operate with incalculable power in suppressing quackery and extending the benefits of honorable and useful dental practice to all orders of society.

Inasmuch as the American Journal of Dental Science is the organ of the Society, in the propagation of the true principles of the science of dental surgery, we call upon our brethren, in all parts of the country, to contribute sedulously to its pages all such articles as may convey useful instruction to any of its numerous readers.

The successive volumes of this work convey to posterity a history of our art in the middle of the nineteenth century, and with it the names and memory of those who nobly sacrifice, upon the altar of the public good, the private advantages of isolated selfishness. There can be little doubt, that some of the distinguished individuals who have aided in forming the national Society, and in establishing the Dental Journal as a vehicle of practical information, have thus waived the privilege of employing the knowledge of their art, which they had gained by extensive travel, by long experience, and unremitted efforts for personal aggrandizement. In this they have not, indeed, experienced the peculiar emotions of a dentist residing in one of our large cities, who recently inquired of a member of the American Dental Society, "*whether admission to membership would put any money into his pocket;*" and added, that "*if it would, he should be inclined to become a member.*" Noble and exalted sentiment! worthy of those numerous *Dii minorum gentium,* by whose presence the profession of dental surgery has become so justly distinguished.

But as we have already intimated, the time is rapidly approaching when public opinion will correct the existing evil of an excess of practitioners in our art. That there will always be a few quacks to act as a foil to set off, by contrast, the beauties of genuine art, there is no reasonable doubt. But the majority of those individuals who have rushed into dental practice, unprepared and uncalled, during the recent stagnation in most of the common branches of industry, will soon find that their golden dreams of wealth and greatness, are not likely to be realized in dental practice, and they will therefore prudently betake themselves to various callings for which they are better qualified both by nature and education.

The experience of the past ten years has shown, that, among the hundreds and thousands who have gathered themselves together to what they regarded as the golden harvest of dentistry, ripe and ready for the sickle; only a very small number, perhaps five in a hundred, will ever be able to attain to either eminence or usefulness in operations on the teeth. These operations require a rare and peculiar assemblage of talents, possessed only by a few. As well may every strippling who can raise a pair of curled mustachios, or bushy whiskers, expect to become an accomplished vocal musician, without either ear or voice, as a successful dental surgeon without the necessary talent and the proper education.

We say, therefore, that the time has come when we begin to perceive a manifest *reaction* on the subject of rushing unprepared into dental practice; and in five more years, if the American Society of Dental Surgeons, and their Journal, continue as they have done, to correct the public sentiment, the only road to success in this department of industry and talent, will be a thorough education, and a patient apprenticeship with the eminent practitioners of the art.

B.

### THE WAY SOME PERSONS BECOME DENTISTS.

"A man must serve his time to every trade
Save censure, critics are already made."

This may have been true once, but the poet evidently was no prophet: he did not foresee the day when men should make themselves dentists by a simple act of volition, as the celebrated Jew physician, who gained great reputation in London, made himself a doctor. He was out of money, out of employment, out of patience, and out at the elbows,—suddenly he exclaimed, "I am a doctor;" his education was completed, and in a little while he was in full practice. How many dentists have as suddenly willed themselves into business, by this short-hand method of professional induction, we cannot tell.

Fire in each eye, and forceps in each hand,
They swarm, and cheat, and swagger round the land.

Give one of these gentry a pot of mineral amalgam, and a few leaves of tinfoil, and it will be strange if he does not pick the pockets of a neighborhood. Yet, after all, this must be a bad business: people soon discover that they are cheated; practice falls off, as fast as it came on, and the great dentist, who makes bad teeth to be better than good ones, and fills cavities with indestructible (Cornwall) silver or self-adapting paste, is obliged to leave the scene of his miraculous performances, with more despatch than dignity. This routine of operations is performed again and again, until there remains no place where the man is not known, and no place to which he can go; those that know him best, and are most anxious to see him, are precisely the people whom he desires with all his heart never to meet again.

But there is another class of dentists, who must by no means be confounded with the vagabond order above mentioned, and yet who are very unfit to practice the surgery of the mouth. These are well meaning people, who, either from ignorance of what is necessary to fit them for the profession, or from inability to procure proper instruction, commence practice with scarcely the elementary knowledge of the dental art. They can file a tooth, and perchance extract one. They can paste up a cavity, or fill a hollow grinder with tin or even with gold, but not in such a manner as to secure its preservation. They may even have a sufficient idea of the business to insert, in a bungling way, a pivot tooth. But beyond these rude mechanical efforts, they know nothing, until they acquire experience by practice,—that is to say, until they have committed every sort of blunder so often, and with such painful conseqeunces, as to make it hard for them to err in the same way again. Of this kind of dentists there are great numbers, and greater numbers still are daily joining their ranks.

But there is another class of dental surgeons, very different from those above mentioned. These are the men of science; the men who understand the great principles of medical treatment, who know that the mouth is an integral and most important part of an exquisitely organized whole, and who apply the science of surgery to this one part, not as being detached from the body, but as most closely connected with it. Few of the most skilful surgeons in the country could perform, with excellence, an ordinary dental operation; few could properly pronounce, at a glance, upon the nature and extent of the diseases of the teeth: how then can an uneducated man expect to do this? If the profoundest knowledge of surgery, and constant practice in it, does not make a man a dentist; if even the most skilful surgeon must study the diseases of the mouth and their treatment as a speciality, what strange presumption is it in an uneducated man to undertake the practice of an art, so important and so difficult.

It must be repeated until it obtains the currency of a proverb, that dentistry is a department of surgery, and that the man who would practise it properly must prepare himself by diligent study and careful mechanical operations.—*Balt. Ed.*

*Cutlery.*—Some eighteen months or two years ago we took occasion to notice the high state of perfection, American cutlers had brought the manufacture of surgical and dental instruments, and to contrast those manufactured in this country with those obtained from Europe. We are fully convinced that nothing is now to be gained by sending to England for either dental or surgical instruments. That the European manufacturers greatly excel the American in many articles of cutlery, we do not doubt, but the reason of this we believe to be principally owing to the indisposition on the part of the people of this country to patronize home manufacturers. As for example, not one in a hundred can be found to believe that a pen-knife is worth having, that has not the name of Rogers upon its blades. Until very recently, we were among the number that entertained this opinion, but a few weeks since having occasion to procurc a pocket knife, and after having visited several establishments where such articles are usually kept for sale, without being able to please ourself, we at last stepped into the store of Mr. Samuel Jackson, manufacturer of dental and surgical instruments, Baltimore, where we supplied ourself with, not only one of the most beautiful, but with the very best knife we ever had. It has four blades, and for keenness, delicacy and durability of edge they surpass any of Rogers' we have ever used.—*Balt. Ed.*

*Lithodeon.*—We have frequently expressed our opinion with regard to the value of the *amalgam of mercury and silver,* as a preparation for filling teeth. No matter by what name it may be designated, whether by *lithodeon, mineral paste,* or *royal mineral succedanium,* or with whatsoever it may be combined, we do not believe it can be used with impunity, and we rejoice to know that we are not alone in this opinion. We believe that it is entertained by all who have noticed the effects that result from its use, which are always hurtful, but more so sometimes, than others. The employment of this article for filling teeth, was a subject of discussion at the last meeting of the American Society of Dental Surgeons, and on motion, it was pronounced, without a dissenting voice, to be mal-practice; and at a meeting held in Philadelphia, two years before, this same association expressed a similar opinion concerning the use of this deleterious preparation. But, notwithstanding, we now and then hear of its being occasionally employed by some members of the profession, and most of whom, if they were aware of the pernicious consequences that resulted to their patients from its use, we have confidence to believe, would at once abandon the practice. That many have used it without being aware of its mischievous effects, there can be no doubt, but a moment's reflection ought to be sufficient to convince any one acquainted with the properties of one, and the principal ingredient, of which this amalgam is composed, that if it was not actually hurtful to the teeth, it, at least must, of necessity, be to the soft parts and fluids of the mouth.

Residing as we do in the midst of a community which has suffered much from the use of it, our opportunities for observing its effects, have been ample, and, as we said on another occasion, we do not recollect to have ever met with a case where several teeth in the same mouth, or even one very large cavity had been filled with it, where it had not obviously been productive of injury. A tooth may sometimes be so filled with it as to lead a person to suppose the disease arrested, but the filling always oxydizes more or less rapidly, and the decay in the organ still progresses, though it may not for some time become apparent to the patient. And when it is not productive of immediate observable injury to the teeth, it seldom, if ever fails to vitiate the secretions of the mouth and to excite in the alveolo-dental periostea and margins of the gums around those which have been filled with it, a chronic inflammation, causing a gradual wasting of their sockets and a loosening of the organs. The inflammation thus induced, oftentimes extends to the gums and sockets of all the other teeth, and sometimes to the whole of the mucous membrane of the mouth and to the glands and adjacent muscles.

Salivation often results from the filling of teeth with this preparation. A number of cases have come to our own knowledge, and in a former number of the Journal we gave an account of one where this had happened. We now beg to subjoin a description of two others, which very recently came under our observation.

Mr. K., a gentleman of about thirty years of age, of a sanguinous and rather full habit, and slightly disposed to scorbutus, had, about four months ago, three large cavities in his teeth—one in the first, and one in the second inferior molares, and one in the first superior, all of the left side, filled with what a country dentist who performed the operation, called *lithodeon.* About two weeks after the operation had been performed, the teeth that had been filled, began to be tender and sensitive to the touch. These symptoms gradually increased and were followed by a slight protrusion or elongation of the teeth, and ultimately by soreness of the gums, a copperish taste in the mouth, an offensive breath and viscidity and increased discharge of saliva. To these succeeded a contraction of the temporalis and masseter muscles, clinching his jaws so firmly that they could not be forced asunder more than a quarter of an inch, and it was in this condition, that, he, about seven weeks ago, applied to us for professional aid.

Having been made acquainted with the history of the origin and subsequent progress of the disease, we at once proceeded to remove the fillings from his teeth, intending to extract the diseased organs as soon as the muscles of his lower jaw should become sufficiently relaxed to admit of his mouth being opened wide enough for the introduction of a pair of forceps. We next directed him to take an aperient every other day for a week, and to gargle his mouth in the mean time, half a dozen times a day, with sage tea, borax and honey. The soreness in his teeth and gums gradually subsided; the discharge of saliva diminished in quantity and became less viscid; at the expiration of a week, the aperients were discontinued, and a decoction of the inner bark of young green white oak, substituted for that of the sage tea, borax and honey. In two weeks he was able to open his mouth, and the soreness in the teeth that had been filled, having nearly subsided, we determined not to extract them at once, and in addition to the astringent gargle, we directed him to cleanse his teeth thoroughly four times a day with a brush and floss-silk, as recommended by Dr. L. S. Parmly. In one week more, the soreness in the diseased teeth having entirely subsided, we filled them with gold, and up to the present time, four weeks, they have remained perfectly free from even the slightest uneasiness.

Comments upon this case are unnecessary. It speaks for itself, and proves beyond doubt, that it was the result of the presence of the mercury in the fillings that had been placed in the three molar teeth.

The subject of the other case was a young gentleman about twenty-one years of age, of a scrofulous and irritable habit of body. In the early part of last summer, he had both of his inferior dens sapientiæ, and three superior molares filled with what was called a *mineral paste,* but which was essentially the same article that had been employed in the other case. The symptoms that succeeded the operation in this case, were similar to those of the other, except that the muscles of the lower jaw were not sensibly affected. The teeth that had been filled, and afterwards all the others, became sore and apparently considerably elongated. The gums were swollen and exceedingly sensitive; the whole of the mucous lining of the mouth was inflamed and red, the breath had a very offensive odor, and the saliva was viscid and very abundant. In addition to these symptoms, an abscess had formed in the alveolus of the left dens sapientiæ, which was in a necrosed condition, and in one of the superior molares that had been filled

These symptoms did not manifest themselves all at once, but came on gradually, and as they continued, became more and more aggravated, until finally, about three months after the operation on his teeth, they excited so much alarm, that he was induced to consult us upon the subject. The treatment pursued in this case, was at first similar to that adopted in the one just described. The mercurial fillings and the teeth, in the sockets of which abscesses had formed, were removed, his mouth directed to be gargled half a dozen times a day, and his bowels to be kept open with aperients. This treatment was continued for ten days, when the aperients were omitted and the gargles substituted as before, for a decoction of oak bark. The saliva had by this time become less abundant and less viscid; the breath, however, still continued offensive, and not much improvement was observable in the condition of the gums and alveoli. At the expiration of fifteen days, the alveolus of the dens sapientiæ that had been extracted, was found to be loose as well as that also of the tooth anterior to it; these, together with the contained tooth, were removed. The teeth in the anterior part of the mouth gradually became firm, but the right inferior dens sapientiæ and one of the remaining upper molares that had been plugged, continuing very loose, we extracted them. About a week after the other molaris which had become firm in its socket, was filled with gold, and has since remained free from pain.

The two foregoing cases are presented to our readers, because we believe the facts connected with them should be known to the profession generally, and with the hope, that a knowledge of them may serve to deter such as may be in the habit of occasionally employing this poisonous compound for filling teeth, without being aware of the deleterious effects that are liable to result from its use. They are seldom, it is true, as bad as those produced in the cases just described, but they are always hurtful, and more so in proportion as the individual is susceptible to the action of mercurial medicines.

At the commencement of our remarks we alluded to the action which the American Society of Dental Surgeons had had in relation to the employment of this noxious amalgam, but we forgot to state, that this association had appointed a committee at its last meeting to collect facts upon the subject and report the same to Dr. Westcott of Syracuse, N. Y., to be by him laid before the Medical Society of Onandagua County of said state, where, at its next meeting, a further investigation of the matter is expected to take place. If that committee discharges its duty, there will not be wanting facts to prove to the satisfaction of all unprejudiced and *even prejudiced* minds, that an *amalgam of mercury*, and *silver* is a preparation that should *never, under any circumstances*, be employed for filling teeth. H.

*Our late Coadjutor.*—Scarcely had we expressed our satisfaction at the appearance of a new Dental Journal, in the British metropolis, before we are called upon to perform the unpleasant duty of recording its demise. The second number of the "British Quarterly Journal of Dental Surgery," edited by J. Robinson, Esq., was received in this country during the past summer, and, as we learn by letter from its editor, was the last of the series. Whether the indisposition to sustain the British Journal on the part of our English professional brethren, arose from their kind intention of giving their patronage to the American Journal and Library of Dental Science, we have no means, at present, positively to determine; but, be that as it may, we feel very thankful for the countenance and support which some of their number have given to our undertaking from its commencement.

But in spite of the temptation which we feel, to indulge the hope that our transatlantic brethren regard our work as well calculated to meet the wants of the profession, in all countries where the English language is spoken, we sincerely regret that our short lived cotemporary should not have been indulged with a more protracted existence. We had hoped that the spirit and enterprize of the profession would have sustained a Dental Journal both in England and America. But if we are convinced of our mistake, we hope we are justified in believing that at least one periodical, devoted to the dental art, will advance to a good old age,—not less than "three score years and ten." If that should be the unprecedented good fortune of the American Journal and Library of Dental Science, we shall have no occasion to blush, in the persons of our children, for having commenced the work. B.

*Salivary Calculus.*—In another part of the present number of the Journal, is a paper, read by M. Mandl, at the French Academy of Sciences, on the "*tartar of the teeth*" in which a new theory on the manner of the formation of this substance, is promulgated. The other theories upon the subject, M. Mandl disposes of in a very summary way, by simply declaring them to be hypothetical and unsanctioned by "direct experience." But while he thus unceremoniously condemns all former explanations concerning the source from whence this earthy matter is derived, as erroneous, it will be necessary for him to adduce facts more conclusive than any which he has yet done, to establish the correctness of his own.

Admitting the tartar of the teeth to be composed, as M. Mandl asserts, of the skeletons of dead infusoria, it by no means follows, that the earthy matter, of which they are principally composed, is not derived from the saliva. On the contrary, that it is derived from this fluid, is rendered conclusive, by the fact, that this earthy deposition, is always first found upon the teeth, immediately opposite the mouths of the salivary ducts, and that it is comparatively seldom that it is met with on the other teeth. It is often, too, found within the very mouths of these ducts.

If the theory of M. Mandl, were correct, tartar when found in the mouth would be deposited upon all the teeth alike. The fact stated by him, that infusoria may be seen, in mucous matter, found around and between the teeth, when diluted with distilled water, proves nothing at all, for it is well known that the tartar of the teeth is always more or less mixed with the mucous secretions of the mouth, and if the infusoria have their origin in this fluid, their presence here, may be thus accounted for.—*Balt. Ed.*

*Eleazar Gidney, Esq., Surgeon Dentist.*—Who, for a number of years, has resided in Manchester, England, where he has enjoyed an extensive and lucrative practice, returned, from a short visit which he made to this country, on board the Great Western, the latter part of October last. The confidence which American dentists command abroad, and even in England and France, ought certainly to be gratifying to the pride of the profession in this country. Mr. G. as is well known, is a native of and practiced his profession for many years in the United States, and had acquired a high reputation for skill, previously to his going to England. He was, therefore, deserving of all the confidence and patronage with which he has been honored in that country.—*Balt. Ed.*

*Gossip with our Profession: by the Junior Editor.*—He who will give distinct, simple, and easily understood names for all the normal elevations and depressions, aspects and surfaces of the human teeth, will do the profession a service. The nomenclature once generally adopted, would make the reporting of cases much more easy to the reporter, and the report would be much more easily understood. Every dentist knows how difficult it is to describe his operations, even to one of his own profession. * * * * We often hear it said, "If I cut down to the alveolus, all around a tooth, I cut all that can be cut," &c.; but close and long continued observation, and much practice, have taught *us*, that the strongest attachment that can be cut, is generally *not* cut—even though the operator do "cut down to the alveolus all around." As a proof, observe the little glistening fibrous mass of flesh upon one or two sides, near the crown, of nine-tenths of the teeth you extract, after "cutting down all around" them. *We* cut thus: suppose we are to extract an inferior molar; we take an instrument with a blade like that of a penknife—narrow, straight on the back, tapering to a point, and very sharp. With the handle elevated some thirty or forty degrees, we insert the point, *with the back down,* through the gum about a line from its margin, outside the arch, opposite the anterior side of the tooth, and pass the blade through toward the tongue, with the point of the blade in the socket of the tooth. We do the same with the opposite side of the tooth; and then with a very narrow curved lance, we *cut down* in the usual manner on the exterior and interior sides. We make no pretensions, however, to cutting any *ligament,* or any other *generally* very strong attachment, by our method. Since a most unpardonable deception by the pretended discoverer of the ***ligamentum dentis*** upon *ourself,* (the immediate consequences of which were such as we hope never to feel again, and the ultimate consequences we are yet in great fear of,) an operation performed to convince us of the existence of that ligament; our estimation of both the discovery and the discoverer has sunk considerably ***below*** zero—the point *at* which it stood before. * * * * In the earlier years of our practice, it was our misfortune (and our patient's also) to have the operation of engrafting teeth upon fangs frequently followed by pain, swelling of face, &c. We tried all the harmless prescriptions for prevention, or remedy, that we could hear of, but without satisfaction. A groove in the side of the *dowel,* (not *pivot,* gentlemen,) would afford an exit for the matter collected, or rather *deposited,* in the fang, and thus prevent the formation of an abscess at its apex: but we have become convinced by some years' practice in our *present* treatment of fangs, preparatory to engrafting crowns upon them, that this matter in the fang is the cause of the after-trouble in a vast many cases. If the nerve be not already dead in the fang, we destroy it *entirely;* and, in all cases, plug the fang with gold, from the apex to the end of the dowel. This prevents the stagnation and horrible fetor of animal matter within the fang: and, in our practice, not only prevents subsequent disease almost always, but will put an end to disease already existing in a great many cases where the common practice has been followed. * * * * The question has been often asked us, "have you used platina plate, and if so, do you approve of it?" We have used it. In some cases where we wished a purer material than gold solder, we have used platina plate, and, instead of solder, have used gold coin, which will *flow* upon platina very beautifully. We confess, however, that platina has weighty objections; it is heavier and less elastic than gold, and not so easily wrought. The difference in cost between the two metals, we have found to be more than counterbalanced by the difference in the labor which they respectively require to work them. * * * If there be any of our profession who have not yet used the grinding wheels made of shell-lac and emery, (such as are sold at Stockton's establishment,) we would say to such *try them:*—and if they *do* try them, they will give their old grind-stones to the children to play with. * * * * We take some little credit (with all due modesty) for pointing out the *use* of the *rugæ* in the anterior portion of the *roof* (the term is expressive) of the mouth. They aid *very materially* in producing all the hissing sounds—as of c, s, z, &c. &c., by allowing the tongue to rest steadily *against* them while they admit a passage of air *between* them. And now for the practically valuable results of the discovery: when we must use wide dental plates, if these *rugæ* cannot be stamped up, they should be soldered on. * * * * There are unmentionable associations connected with the *syringe* in its common shape—a serious objection to its use about the mouth. The objection is removed, by so altering its form that it may not be recognized as a

***syringe*** by the patient. We have one of those pear-shaped gum-elastic bottles, or sacks, such as are sold by the apothecary, which will contain, perhaps one-fourth of a gill: in the neck of this is fastened one end of a silver tube, two inches long and one-eighth of an inch in diameter; to the other end are fitted several thin tubes, of different curves and sizes. When we would use the instrument, we collapse the sack by pressure in our hand—insert the free end of the larger tube into water, and unclose the hand—when the sack will regain its former shape, filled with water. The small tube we would use, is now inserted, and the syringe is ready for use—the water being forced out at pleasure by merely closing the hand that applies the instrument. When neatly made it is far preferable to those in common use, on account of its convenience, appearance and its simplicity; and it does not cost one-fourth as much. * * * * Will some one furnish us with a specimen of "internal caries of the teeth"—not caries around the cavity of a dead nerve, but such "internal caries," as is so minutely described by some of our "popular authors?" We never saw a case, and, with all respectful deference for the opinions of said authors generally, and, for that of Professor Dunglisson particularly, who says, (in his review of Robinson's excellent work,) that caries *always* commences *within* the tooth; we doubt that such caries ever existed. We feel pleasure in adding that there are *many* others of our profession, who are of our way of thinking in this matter. * * * * We have in our possession a curiosity, entitled an "Essay on the Teeth." Its author says the decay found in the grinding surfaces of the teeth, is the consequence of *grits* lodging in the depressions, and grinding holes through the enamel!—and that the decay found upon the sides of the teeth is accounted for by the *fact* [!] that at those places there is a deficiency of enamel, the teeth being *in contact,* when the enamel is deposited!!! The profundity of his dental research strikes us with inexpressible astonishment. Why, the man nearly equals that old Dutch anatomist, who says the roots of the first teeth, are the seeds from which the second grow! M.

